# Is There a Role of Autophagy in Depression and Antidepressant Action?

**DOI:** 10.3389/fpsyt.2019.00337

**Published:** 2019-05-15

**Authors:** Nils C. Gassen, Theo Rein

**Affiliations:** ^1^Department of Psychiatry, Bonn Clinical Center, Bonn, Germany; ^2^Max Planck Institute of Psychiatry, Munich, Germany

**Keywords:** autophagy, depression, antidepressant, stress, FKBP51 signalling

## Abstract

Autophagy has been recognized as evolutionary conserved intracellular pathway that ensures energy, organelle, and protein homeostasis through lysosomal degradation of damaged macromolecules and organelles. It is activated under various stress situations, e.g., food deprivation or proteotoxic conditions. Autophagy has been linked to several diseases, more recently also including stress-related diseases such as depression. A growing number of publications report on the role of autophagy in neurons, also referred to as “neuronal autophagy” on the one hand, and several studies describe effects of antidepressants—or of compounds that exert antidepressant-like actions—on autophagy on the other hand. This minireview highlights the emerging evidence for the involvement of autophagy in the pathology and treatment of depression and discusses current limitations as well as potential avenues for future research.

## Depression Is a Prevalent and Severe Disease

Worldwide, depression is one of the most frequent clinical conditions and the leading cause of disability affecting more than 300 million people of all ages, according to World Health Organization (WHO) statistics (http://www.who.int/news-room/fact-sheets/detail/depression). Depression is characterized by a cluster of symptoms that include depressed mood, fear, feelings of worthlessness, loss of energy and interest, reduced responsiveness to pleasurable stimuli, lack of appetite, cognitive impairment, and sleep disturbances (1). A high percentage of seriously depressed patients receive no appropriate treatment, even in developed countries (2). Suicidal ideation is a further characteristic of depression and up to 15% of severely depressed individuals commit suicide. Depression represents also a major independent risk factor for other diseases like cardiovascular disease, dementia, diabetes, and osteoporosis (3, 4).

The high complexity of this mental disorder accounts for the difficulties in elucidating its molecular underpinnings. Overall, it has been increasingly accepted that a multitude of factors ranging from genetic predisposition to environmental challenges contribute to the pathophysiology of depression. In addition to the analysis of specific targets, research efforts increasingly resort to screening platforms to probe the genome, epigenome, etc. in an unbiased way. Examples of the major specific systems under investigation are monoaminergic, glutamatergic, and stress hormone systems, neuropeptides as modulators of the neuronal cell function including neurogenesis, neuronal morphology, and intracellular signaling pathways.

In genetics, huge efforts produced intriguing results; however, the field is haunted by the lack of consistency and reproducibility [for a recent review, see Ref. (5)]. Thus, increasingly large cohorts are investigated, and meta-analyses are employed to probe several hundred thousands of individuals (6). Nevertheless, not the least due to the difficulties to move from gene association to molecular mechanism, hypothesis-driven approaches continue to be pursued intensely.

Monoamine deficiency was the first hypothesis unfolded over several years, tracing back more than half a century, and probably is the most influential one (7, 8). It postulates lack of monoaminergic neurotransmitters and thus impaired synaptic neurotransmission as cause for depression; several newer antidepressant drugs were developed based on this hypothesis. Other examples include glutamatergic dysfunction and the corticosteroid hypothesis of depression (7, 9). A vast array of studies supports the link between the stress hormone system and depression (9, 10). More specifically, impaired corticosteroid receptor function has been suggested to result in inappropriately high secretions of corticotropin releasing hormone (CRH), vasopressin, adrenocorticotropin, and cortisol (9). A role of autophagy in depression is a more recent hypothesis put forward (11), which can be viewed as one of the ramifications of the stress response as outlined below.

## Autophagy Is a Cellular Homeostasis Process and Part of the Stress Response

Autophagy is a pivotal process to ensure homeostasis of cells, and thus of tissues and the organism, in physiological as well as pathological situations (12, 13). This highly conserved mechanism leads to the degradation of damaged cytosolic proteins, aggregates, organelles, and also pathogens through a step-wise process. The basic mechanism is detailed in several excellent reviews (13–15), so it is described here only briefly: Autophagy involves a series of autophagy-related genes (ATGs), originally identified in yeast. Initially, membrane material is excised, most likely from the endoplasmic reticulum, giving rise to a membrane sac that is further expanded to form a double membrane vesicle called autophagosome. To be degraded material is enclosed into this vesicle; selected additional material can be transferred into the autophagosome. Degradation is achieved upon fusion with lysosomes to form autolysosomes: From the initial isolation of membrane material needed for the formation of autophagosomes to the final fusion step, autophagy involves a number of proteins governing membrane dynamics (16). There are different types of autophagy, with macroautophagy being the most commonly described one (15); this review only deals with macroautophagy, because research on the emerging subject of neuronal autophagy did not yet aim at specifying the type of autophagy.

The crucial physiological role of autophagy is reflected in its links to several diseases and the increasing efforts to exploit this process for pharmacological intervention (17–21). Initially, autophagy was identified as response to calorie restriction to maintain energy homeostasis (22). Today, several pharmacological and environmental factors are known to induce autophagy, in particular various kinds of stressors (13, 17). Thus, autophagy is an important facet of the stress response, and like the stress response in general, autophagy is a beneficial process, but excess activation can be detrimental under certain conditions (23, 24). For example, apoptosis (often referred to as Type I cell death) and autophagy are considered mutually exclusive (13). Others debate this exclusiveness and argue that excessive autophagy can cause type II cell death characterized by the formation of large autophagic vacuoles (25, 26).

Chronic stress in mice, which frequently is used to model depression (27, 28), also has been reported to enhance autophagy [for recent examples, see Refs. (29, 30)]. The observation that a further increase in autophagic markers goes along with the reversal of the behavioral effects again argues in favor of autophagy being a beneficial component of the stress response in general (13, 30). Nevertheless, evidence also has been provided for a role of autophagy induction for depressive-like behavior and cognitive impairment induced by prenatal stress (31). Very recently, inhibition of autophagy was shown to attenuate the induction of depressive-like behavior by ecstasy in rats (32).

## Autophagy in Depression: Evidence From Disease and Disease Model Studies

In human, a study using a small sample size found elevated expression of autophagy genes in blood mononuclear cells from individuals suffering from major depression in comparison to healthy controls (33). Similarly, decreased mRNA expression of AKT1 and mTOR was found in individuals with short-term bipolar disorder compared to healthy controls (34), which might lead to the induction of autophagy. Similarly, a post-mortem study revealed compromised mTOR signaling in the prefrontal cortex in major depressive disorder (35). How could this be reconciled with the observation that enhanced autophagy response in blood mononuclear cells to *ex vivo* antidepressant treatment predicts clinical treatment success (36)? Similar to the stress response in general, autophagy is a beneficial response up to a certain limit, so we hypothesize that this adaptation might be insufficient in some (disease) cases and needs further boosting through various kinds of treatments.

Short-term calorie restriction, one of the most efficient inducers of autophagy (22), has been reported to have antidepressant effects in human and antidepressant-like effects in mice, while the effects of long-term calorie restriction are controversial (37). Likewise, physical exercise has been shown both to enhance autophagy (38) and to reduce depressive symptoms in human (39). Nevertheless, given the plethora of effects of both calorie restriction and exercise, these studies only provide a rather vague support of a potential link between autophagy and depression.

Studies more directly documenting a link of autophagy to psychiatric disease mainly were performed with animal models, with all the debated limitations that come with animal models that try to replicate aspects of depression (27). Maternal separation (40) increased autophagic markers in the prefrontal cortex, but not in the hippocampus (41). This is mimicked by the differential effect of corticosterone in primary astrocytes from these brain regions (42), while another study found that prenatal stress significantly elevated autophagy markers in the hippocampus of male offspring (31). On the other hand, signs of decreased autophagy also have been reported in depression-relevant animal models. For example, chronic unpredictable stress decreased autophagic markers (43, 44). LPS as well as unpredictable chronic mild stress induced depression-like symptoms in rodents along with reduced expression of autophagic markers (45, 46). Furthermore, inhibition of the autophagy initiator Beclin1 (47) induced depression-like behavioral changes in mice (48). Thus, no consistent picture of enhanced or reduced autophagy in depression yet emerges from animal models. Further, it is difficult to conclude about functional autophagy, as flux assays or determining turnover of long-lived proteins is complicated to perform in mice.

## Autophagy in Depression: Evidence From Treatment Effects

Given the scarcity of studies on disease correlation, the hypothesis that autophagy is involved in depression mainly is based on the effects of antidepressants on autophagy. One of the earliest hints for a role of antidepressants in autophagy was the observation of autophagy-associated structures in the cytoplasm upon treatment of cells with the tricyclic antidepressant clomipramine (chlorimipramine) (49). This phenomenon could be caused by either induction of autophagy or blocking the autophagy flux, thus actually blocking functional autophagy. It should be noted here that the conclusion of active autophagy often is based on the mere appearance of autophagic markers, which is not correct in the absence of experiments assessing the autophagic flux or turnover of long-lived proteins (50). Employing appropriate experiments, it was shown later that desmethylclomipramine, the active metabolite of clomipramine, interferes with the autophagic flux and thus functional autophagy (51). In contrast to the effect of clomipramine, another tricyclic antidepressant, amitriptyline, was found to increase autophagy in primary neurons and astrocytes, similarly to the selective serotonin reuptake inhibitor citalopram; however, the selective serotonin and noradrenaline reuptake inhibitor venlafaxine did not alter autophagy (52, 53). Thus, it appears that antidepressants diversely impact functional autophagy, possibly also in a cell-type-dependent manner.

Conspicuously, the canonical autophagy inducer rapamycin has been found to exert antidepressant-like effects (54, 55), emphasizing the role of the mTOR pathway (56). Conversely, several other established antidepressants and compounds that are reported to exert antidepressant-like effects were shown to modulate autophagy in various experimental models. Among the established antidepressants are the tricyclic antidepressants desipramine, nortriptyline, and imipramine, the tetracyclic antidepressants maprotiline and mianserin, the noradrenergic and serotonergic antidepressant mirtazapine, the selective serotonin reuptake inhibitors fluoxetine (Prozac), sertraline, and paroxetine, the serotonin-norepinephrine reuptake inhibitor desvenlafaxine, the atypical antidepressant agomelatine, lithium [for a review, see Ref. (57)], and the anticonvulsant valproic acid. Further drugs with both antidepressant-like effects and impact on autophagy include trehalose, hypericin, which is one of the principal components of Saint John’s wort, Salvianolic acid B, rosiglitazone, silibinin, dapsone, geldanamycin, α-tocopherol, and extracts of Euryale ferox Salisb (see Table 1 for more details and citations). Of note, also electroconvulsive therapy, which particularly is used for severe or treatment-resistant depression (58), was reported to enhance autophagy (59).

**Table 1:** Overview of the various autophagy-impacting compounds that are used as antidepressants or reported to exert antidepressant-like effects in animal models.

Compound/Antidepressant	Experimental system	Results, autophagic markers	Flux, LLP	Citation
Clomipramine*, Desmethyl-clomipramine	Human glioma cells	Autophagy-associated structures	no	(49)
	HeLa Cells, ATG5^-/-^MEFs	LC3BII/I up, increase in DM structures, flux blocked, LLP degradation down	yes	(51)
Amitriptyline*	Primary rat astrocytes and neurons, ATG5^-/-^MEFs	Increased autophagy (LC3BII/I, Beclin1 up)	yes	(52)
	Mouse stress model, patient blood cells, HEK cells, rat cortical astrocytes	ATG12, LC3II/I, Beclin1, pAkt1 and VPS34 were up, increased flux	yes	(36)
	Corticosterone-stressed mice	Increased autolysosomes, affects pBeclin, pULK, increased p62	no	(48)
Citalopram*	Primary rat astrocytes and neurons	Increased LC3BII/I and Beclin1	no	(52)
Venlafaxine*	Primary rat astrocytes and neurons	No effect	no	(52)
Desipramine*	C6 glioma cells	Inhibition of mTor pathway, increased Beclin1, LC3, autophagosomes	no	(60)
	L929 cells ATG7^-/-^ MEFs	Autophagy induction (LC3II/I up, p62 down,	no	(61)
Nortriptyline*	High content chemical screen in HeLa cells	Autophagy induction (LC3II/I, flux)	yes	(62)
Imipramine*	Glioma cells, mouse models of gliomagenesis	Upregulation of LC3II/I, increased flux, more autophagic vacuoles	Yes (cells)	(63)
	THP-1 cells, depressed patients, ATG5^-/-^MEFs	mRNA of LC3 and Beclin1 up, LC3II/I up	no	(64)
	U-87MG glioma cells	Inhibition of PI3K/Akt/mTOR signaling, LC3II/I up	no	(65)
Maprotiline*	Burkitt’s lymphoma cell line	Beclin1 up, more cytoplasmic vacuoles	no	(66)
Mianserin*	THP-1 cells, depressed patients	mRNA of LC3 and Beclin1 up	no	(64)
Mirtazapine*	THP-1 cells, depressed patients, ATG5^-/-^MEFs	mRNA of LC3 and Beclin1 up, LC3II/I up	no	(64)
Fluoxetine*	Human breast cancer cell lines	Upregulation of LC3II/I, Beclin1, ATG5; p62 down	yes	(67)
	Human adipose-derived stem cells, mature adipocytes	Upregulation of LC3II/I, ATG12, SQSTM1, Beclin1, ATG7	no	(68)
	Brain injury in rats	Upregulation of Beclin1, LC3 punctae	no	(69)
	Stress model in rats	Upregulation of Beclin1 and LC3II increased PI3K/Akt/mTOR activity.	no	(43)
	Burkitt’s lymphoma cell line	Beclin1 up, more cytoplasmic vacuoles	no	(66)
Sertraline*	Non–small cell lung cancer cells	LC3II up, increased flux, autolysosome formation	yes	(70)
	AML cell lines	LC3II/I increased	no	(71)
Paroxetine*	THP-1 cells, depressed patients	mRNA of LC3 and Beclin1 up	no	(64)
	Mouse stress model, patient blood cells, HEK cells, rat cortical astrocytes	ATG12, LC3II/I, Beclin1, pAkt1 and VPs34 were up, increased flux	yes	(36)
Desvenlafaxine*	THP-1 cells, depressed patients	mRNA of LC3 and Beclin1 up	no	(64)
Agomelatine^#^	THP-1 cells, depressed patients	mRNA of LC3 and Beclin1 up	no	(64)
Lithium*	ALS mouse model	Increased number of autophagic vacuoles (Beclin1 and LC3)	no	(72)
	Prion-infected cells	LC3II/I and flux increased	yes	(73)
VPA*	Human glioma cell lines	LC3II/I and Beclin1 increased	no	(74)
Ketamine*	Human epithelial cells	LC3II/I and Beclin1 increased	no	(75)
Trehalose	Mouse model of manic-like behaviors	Reduced ratio of p62/beclin1 in the frontal cortex	no	(76)
	Diverse mammalian cells, ATG5^-/-^MEFs	Increased LC3II/I, flux	yes	(77)
Hypericin	Human macrophages	LC3II/I and Beclin1 up, p62 down, only in combination with ultrasound	no	(78)
	Leishmania promastigotes	mRNA of AMPK up, ATGs diversely regulated	no	(79)
Salvianolic acid B	Depression model in rats	Compound restores treatment-induced impairment of autophagy (LC3II/I, Beclin1)	no	(46)
Rosiglitazone*	Depression mouse model, N2a cells, primary neurons	Increases Beclin1, ULK1, LC3II/I, pAMPK, and pAKT1, decreases p62 in stressed mice	no	(45)
Silibinin^#^	Depression mouse model	Decreased LC3II/I	no	(80)
Dapsone*	Cognition-compromised rats	Enhanced LC3II/I and Beclin1, decreased p62	no	(81)
Geldanamycin	Rat model of anxiety and depression	Atg12, Atg7, and LC3II/I increased	no	(82)
α-tocopherol*	Mouse model of depression	Enhanced LC3II/I, pAMPK decreased p62, pmTOR	no	(44)
Euryale ferox Salisb extracts	Mouse model of depression, HT22 cells	Enhanced LC3II/I, pAMPK decreased p62, pmTOR	no	(83)

*Labels drugs approved by the United States Food and Drug Administration. ^#^Approved in the European Union. LLP, assay to determine the stability of long-lived proteins; DM, double membrane; MEF, mouse embryonic fibroblasts; VPA, valproic acid; AML, acute myeloid leukemia; ALS, amyotrophic lateral sclerosis.

Mechanistically, antidepressants appear to address various pathways to impact autophagy. For example, FKBP51, which is a glucocorticoid receptor and stress regulator linked to psychiatric diseases (84–86), has been shown to be required for the effects of antidepressants on both autophagy and depressive-like behavior (36, 87). Another very recent study discovered that the previously reported effects of antidepressants on the acid sphingomyelinase (ASM) (88, 89) trigger a pathway leading to upregulation of autophagy, which is required for the behavioral effects in mice (48). More specifically, this pathway involves the accumulation of antidepressants in lysosomes, where they inhibit ASM. This leads to an increase in sphingomyelin and finally of ceramide in the endoplasmic reticulum. Ceramide, in turn, activates the phosphatase PP2A, which stimulates the kinase ULK, a known activator of autophagy (48).

**Figure 1:**
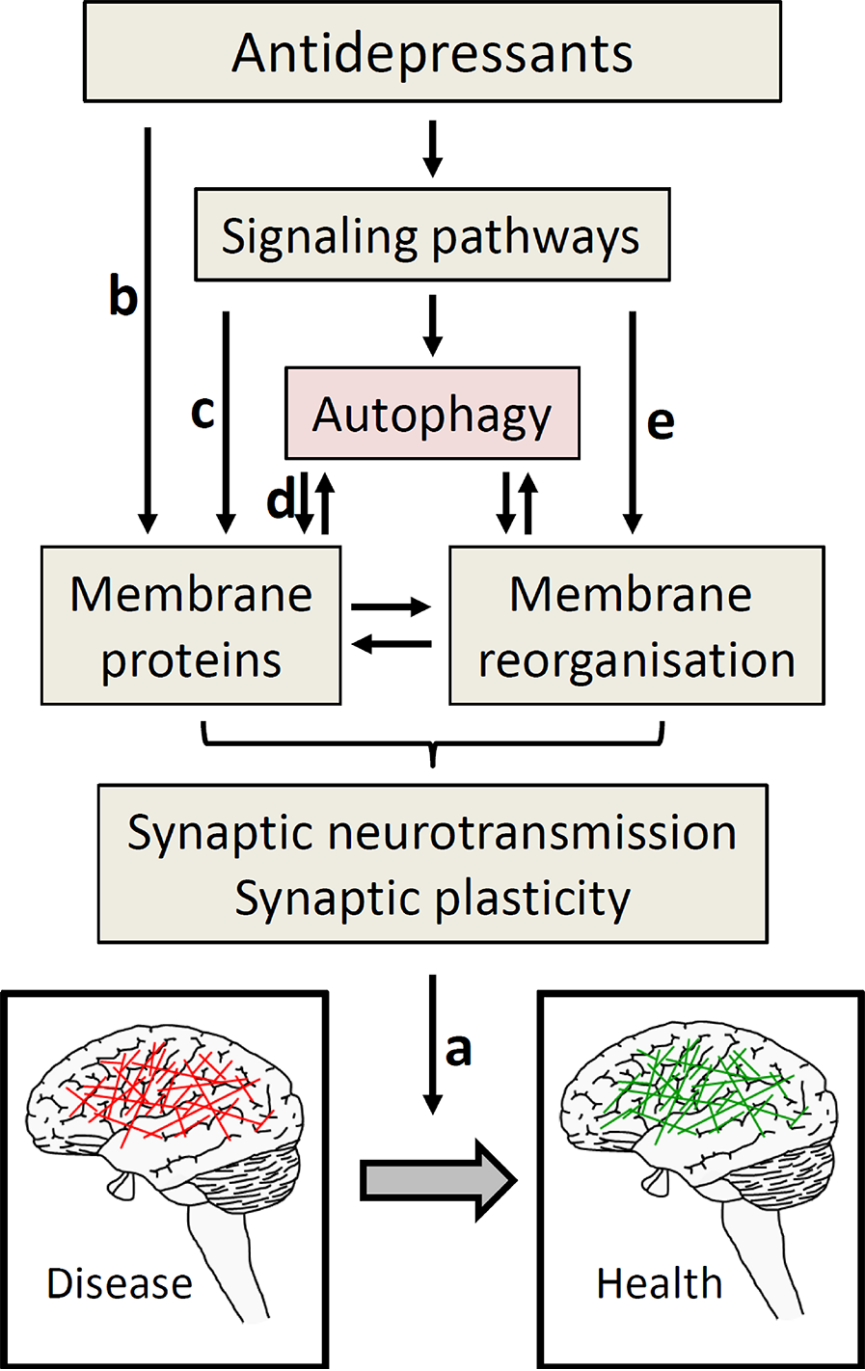
Autophagy as part of antidepressant action. To move from diseased (depressed) to healthy state, ultimately a change in neuronal activity is required (A). To achieve this, several ways of antidepressant actions are proposed including effects on hormonal systems, immune system, and neurogenesis, which all might be intertwined with autophagy (97); this figure focuses on synaptic neurotransmission. The by far most often described effect of antidepressants on synaptic neurotransmission operates through directly blocking neurotransmitter reuptake transporters (part of the membrane proteins, (B). These transporters may also be addressed through signaling pathways that regulate their expression and/or function (C), not part of this review). The role of autophagy in antidepressant action frequently is explained by maintaining protein homeostasis in general, and the functional integrity of membrane proteins involved in synaptic neurotransmission in particular (D). These membrane proteins comprise not only transport proteins, but also, e.g., presynaptic SNARE proteins engaged in neurotransmission. Given the similarity of membrane dynamic processes in autophagy and synaptic neurotransmission, and to reconcile the diverse findings of antidepressant effects on autophagy, we also discussed the hypothesis that antidepressants address pathways that change membrane organization, directly linking to synaptic neurotransmission (E).

Of the pleiotropic effects of the mood stabilizer lithium (90), its autophagy-inducing action does not operate through GSK3β, but by inhibition of inositol monophosphatase (91). Despite first glimpses, overall there is considerable lack of mechanistic understanding of how antidepressants link to autophagy. This is partly due to the incomplete knowledge about the molecular interaction partners of antidepressants. Progress in this direction (92) will help elucidating the molecular connection to autophagy. This may also contribute to sorting the actions of antidepressants, because not everything antidepressants do has to be related to depression treatment. Another long-standing conundrum in understanding how antidepressants work is the observation that clinical effects typically take weeks to become manifest, while known targets like neurotransmitter transporters are affected immediately. It is unlikely that autophagy will offer an obvious solution. Arguably, it contributes to starting a process of neuronal reorganization that ultimately constitutes the transition from disease to health (cf. **Figure 1**). Neurogenesis might be part of this process, as extensively discussed elsewhere (93). In this context, it is intriguing that autophagy increases adult neurogenesis (94, 95); thus, it is possible that antidepressants and lithium operate, at least in part, through autophagy to induce neurogenesis (90, 96).

The vast majority of publications report an increase in autophagy by antidepressants. This is also the case for the fast-acting antidepressant ketamine (75), even though it is known to enhance mTOR activity (98). However, as alluded to above, flux assays are missing in many studies (cf. **Table 1**), which may lead to erroneous interpretation and conflicting results. More specifically, many of these reports merely observed an upregulation of autophagic markers, for example, lipidation of LC3B (i.e., an increase in the ratio of LC3II/I), which is not sufficient to make conclusions about functional autophagy. Overall it appears more likely that antidepressants diversely affect autophagy. Another important issue is the concentration at which antidepressants are administered in experimental models. This concentration typically is in the range of 10 µM n cell culture or 10 mg/kg in animal experiments, sometimes even higher. While it has been reported that similar doses can be reached in the brain (99, 100), effects reached at concentrations based on the results of therapeutic drug monitoring (101) may more closely mimic the clinical situation. For example, paroxetine (used at the therapeutic drug dose of 120 ng/ml = 0.9 µM) and amitriptyline (used at the therapeutic drug dose of 120 mg/ml = 0.37 µM) enhanced the expression of autophagy markers in blood mononuclear cells from depressed patients exposed to these reduced concentrations *ex vivo* (36).

## Conclusion and Outlook

Over the last few years, several studies provided evidence for a link of autophagy to the pathophysiology and treatment of depression. Despite impressive progress, the mechanism is far from being understood. This is not surprising for a complex disease like depression, which poses particular experimental challenges and epistemological limitations, as exemplified by the complex mechanisms linking stress with depressive behavior. The molecular effects of antidepressants ultimately need to produce alterations in the pattern of neuronal activity that underlie the transition between diseased and healthy status (**Figure 1**). This means that some neuronal activity needs to be decreased and some needs to be increased. Interestingly, neuronal stimulation not only induces autophagy (102); increased autophagy also impacts synaptic function. For example, induction of autophagy by mTOR inhibition in presynaptic terminals rapidly alters presynaptic structure and reduces neurotransmission (103). Conversely, loss of autophagy slows down synaptic neurotransmission while gain of autophagy increases it (104). The latter finding has been conceptualized by the function of autophagy in protein homeostasis by removing damaged proteins, in this case those involved in synaptic vesicle exocytosis in particular (104, 105). Intriguingly in a mouse model of learnt helplessness evoking depressive-like behavior, decreased levels of the presynaptic vesicle membrane docking and fusion SNARE protein Snap25a occur along with impaired autophagy; administration of fluoxetine attenuates both these effects (106). The SNARE proteins are important components of the membrane reorganizing machinery at the synaptic membrane, and there is an interdependence between autophagy and synaptic vesicle trafficking (107, 108). In addition, electroconvulsive therapy enhances not only autophagic markers (59) but also the membrane trafficking machinery (109).

In light of the presumably diverse impact of antidepressants on autophagy, enhanced recycling of distinct synaptic proteins by inducing autophagy is unlikely to fully picture the mechanism of antidepressants. Given the fact that autophagy needs the activity of a number of membrane reorganizing and membrane trafficking proteins, we consider it plausible that processes impacting autophagy may also impact membrane reorganizing processes at the synapse, and thus would not require the later steps autophagy (cf. **Figure 1**). In general, these processes could be fast, because they do not necessarily require the synthesis of new proteins. They could limit synaptic neurotransmission when autophagy and neurotransmission compete for additional membrane material; conversely, autophagy would promote neurotransmission if there are shared mechanisms for the generation and fusion of membrane material. Thus, it will be of great interest to learn about the conditions under which autophagy increases or decreases synaptic neurotransmission, possibly in a neurotransmitter-specific fashion.

Experiments employing genetic and pharmacological intervention strategies are needed to finally proof the involvement of functional autophagy in antidepressant action and to disentangle the mechanism including the level of synaptic neurotransmission. More specifically, (conditional) knock-outs of central autophagy genes are available in mice. The high interest in autophagy modulators has led to the discovery of a range of novel autophagy inducers and inhibitors, which can be tested in animal models in depression. While compounds that inhibit autophagy through blocking the fusion between autophagosome and lysosome frequently elicit toxic effects when applied over a long period of time, they might be useful for assessing the role of autophagy in the immediate actions of antidepressants in some test regimes such as the forced swim test. It will also be interesting to learn whether and how antidepressants can be grouped according to their impact on autophagy. This categorization may not follow the pattern of their mechanism so far known. Finally, it should be investigated whether the dose dependency for autophagy induction by antidepressants is the same as or at least similar to the therapeutic doses.

## Author Contributions

TR drafted the manuscript. NG added critical information.

## Funding

This work was partially funded by a NARSAD Young Investigator Award by Brain and Behavior Research Foundation, honored by P&S Fund (to NG, Grant ID 25348).

## Conflict of Interest Statement

The authors declare that the research was conducted in the absence of any commercial or financial relationships that could be construed as a potential conflict of interest.

## References

[1] Belmaker RH Agam G Major depressive disorder. N Engl J Med (2008) 358:55–68. 10.1056/NEJMra073096 18172175

[2] Lepine JP Briley M The increasing burden of depression. Neuropsychiatr Dis Treat (2011) 7:3–7. 10.2147/NDT.S19617 21750622 PMC3131101

[3] Knol MJ Twisk JW Beekman AT Heine RJ Snoek FJ Pouwer F Depression as a risk factor for the onset of type 2 diabetes mellitus. Diabetologia (2006) 49:837–45. 10.1007/s00125-006-0159-x 16520921

[4] Nicholson A Kuper H Hemingway H Depression as an aetiologic and prognostic factor in coronary heart disease: a meta-analysis of 6362 events among 146 538 participants in 54 observational studies. Eur Heart J (2006) 27:2763–74. 10.1093/eurheartj/ehl338 17082208

[5] Shadrina M Bondarenko EA Slominsky PA Genetics factors in major depression disease. Front Psychiatry (2018) 9:334. 10.3389/fpsyt.2018.00334 30083112 PMC6065213

[6] Wray NR Ripke S Mattheisen M Trzaskowski M Byrne EM Abdellaoui A Genome-wide association analyses identify 44 risk variants and refine the genetic architecture of major depression. Nat Genet (2018) 50:668–81. 10.1038/s41588-018-0090-3 PMC5934326 29700475

[7] Hillhouse TM Porter JH A brief history of the development of antidepressant drugs: from monoamines to glutamate. Exp Clin Psychopharmacol (2015) 23:1–21. 10.1037/a0038550 25643025 PMC4428540

[8] Liu B Liu J Wang M Zhang Y Li L From serotonin to neuroplasticity: evolvement of theories for major depressive disorder. Front Cell Neurosci (2017) 11:305. 10.3389/fncel.2017.00305 29033793 PMC5624993

[9] Holsboer F The corticosteroid receptor hypothesis of depression. Neuropsychopharmacology (2000) 23:477–501. 10.1016/S0893-133X(00)00159-7 11027914

[10] De Kloet ER Joels M Holsboer F Stress and the brain: from adaptation to disease. Nat Rev Neurosci (2005) 6:463–75. 10.1038/nrn1683 15891777

[11] Jia J Le W Molecular network of neuronal autophagy in the pathophysiology and treatment of depression. Neurosci Bull (2015) 31:427–34. 10.1007/s12264-015-1548-2 PMC5563719 26254058

[12] Rubinsztein DC Shpilka T Elazar Z Mechanisms of autophagosome biogenesis. Curr Biol (2012b) 22:R29–R34. 10.1016/j.cub.2011.11.034 22240478

[13] Kroemer G Marino G Levine B Autophagy and the integrated stress response. Mol Cell (2010) 40:280–93. 10.1016/j.molcel.2010.09.023 PMC3127250 20965422

[14] Noda NN Inagaki F Mechanisms of autophagy. Annu Rev Biophys (2015) 44:101–22. 10.1146/annurev-biophys-060414-034248 25747593

[15] Galluzzi L Baehrecke EH Ballabio A Boya P Bravo-San Pedro JM Cecconi F Molecular definitions of autophagy and related processes 3687. EMBO J (2017a) 36:1811–36. 10.15252/embj.201796697 PMC5494474 28596378

[16] Noda T Autophagy in the context of the cellular membrane-trafficking system: the enigma of Atg9 vesicles. Biochem Soc Trans (2017) 45:1323–31. 10.1042/BST20170128 PMC5730941 29150528

[17] Galluzzi L Bravo-San Pedro JM Levine B Green DR Kroemer G Pharmacological modulation of autophagy: therapeutic potential and persisting obstacles. Nat Rev Drug Discov (2017b) 16:487–511. 10.1038/nrd.2017.22 28529316 PMC5713640

[18] Levine B Kroemer G Autophagy in the pathogenesis of disease. Cell (2008) 132:27–42. 10.1016/j.cell.2007.12.018 18191218 PMC2696814

[19] Levine B Packer M Codogno P Development of autophagy inducers in clinical medicine. J Clin Invest (2015b) 125:14–24. 10.1172/JCI73938 25654546 PMC4382267

[20] Rubinsztein DC Codogno P Levine B Autophagy modulation as a potential therapeutic target for diverse diseases. Nat Rev Drug Discov (2012a) 11:709–30. 10.1038/nrd3802 PMC3518431 22935804

[21] Rubinsztein DC Bento CF Deretic V Therapeutic targeting of autophagy in neurodegenerative and infectious diseases. J Exp Med (2015) 212:979–90. 10.1084/jem.20150956 PMC4493419 26101267

[22] Bagherniya M Butler AE Barreto GE Sahebkar A The effect of fasting or calorie restriction on autophagy induction: a review of the literature. Ageing Res Rev (2018) 47:183–97. 10.1016/j.arr.2018.08.004 30172870

[23] Maiuri MC Zalckvar E Kimchi A Kroemer G Self-eating and self-killing: crosstalk between autophagy and apoptosis. Nat Rev Mol Cell Biol (2007) 8:741–52. 10.1038/nrm2239 17717517

[24] Fabrizi C De VS Somma F Pompili E Catizone A Leone S Lithium improves survival of PC12 pheochromocytoma cells in high-density cultures and after exposure to toxic compounds. Int J Cell Biol (2014) 2014:135908. 10.1155/2014/135908 24563652 PMC3915898

[25] Booth LA Tavallai S Hamed HA Cruickshanks N Dent P The role of cell signalling in the crosstalk between autophagy and apoptosis. Cell Signal (2014) 26:549–55. 10.1016/j.cellsig.2013.11.028 PMC4054685 24308968

[26] Lockshin RA Zakeri Z Apoptosis, autophagy, and more. Int J Biochem Cell Biol (2004) 36:2405–19. 10.1016/j.biocel.2004.04.011 15325581

[27] Nestler EJ Hyman SE Animal models of neuropsychiatric disorders. Nat Neurosci (2010) 13:1161–9. 10.1038/nn.2647 PMC3750731 20877280

[28] Slattery DA Cryan JF Modelling depression in animals: at the interface of reward and stress pathways. Psychopharmacology (Berl) (2017) 234:1451–65. 10.1007/s00213-017-4552-6 28224183

[29] Woo H Hong CJ Jung S Choe S Yu SW Chronic restraint stress induces hippocampal memory deficits by impairing insulin signaling. Mol Brain (2018) 11:37. 10.1186/s13041-018-0381-8 29970188 PMC6029109

[30] Xiao X Shang X Zhai B Zhang H Zhang T Nicotine alleviates chronic stress-induced anxiety and depressive-like behavior and hippocampal neuropathology *via* regulating autophagy signaling. Neurochem Int (2018) 114:58–70. 10.1016/j.neuint.2018.01.004 29339018

[31] Zhang H Shang Y Xiao X Yu M Zhang T Prenatal stress-induced impairments of cognitive flexibility and bidirectional synaptic plasticity are possibly associated with autophagy in adolescent male-offspring. Exp Neurol (2017) 298:68–78. 10.1016/j.expneurol.2017.09.001 28882411

[32] Shih JH Chiu CH Ma KH Huang YS Shiue CY Yeh TY Autophagy inhibition plays a protective role against 3, 4-methylenedioxymethamphetamine (MDMA)-induced loss of serotonin transporters and depressive-like behaviors in rats. Pharmacol Res (2019) 142:283–93. 10.1016/j.phrs.2019.02.026 30826457

[33] Alcocer-Gómez E Casas-Barquero N Nunez-Vasco J Navarro-Pando JM Bullon P Psychological status in depressive patients correlates with metabolic gene expression. CNS Neurosci Ther (2017a) 23:843–5. 10.1111/cns.12755 PMC6492754 28879683

[34] Machado-Vieira R Zanetti MV Teixeira AL Uno M Valiengo LL Soeiro-de-Souza MG Decreased AKT1/mTOR pathway mRNA expression in short-term bipolar disorder. Eur Neuropsychopharmacol (2015) 25:468–73. 10.1016/j.euroneuro.2015.02.002 PMC5863235 25726893

[35] Jernigan CS Goswami DB Austin MC Iyo AH Chandran A Stockmeier CA The mTOR signaling pathway in the prefrontal cortex is compromised in major depressive disorder. Prog Neuropsychopharmacol Biol Psychiatry (2011) 35:1774–9. 10.1016/j.pnpbp.2011.05.010 PMC3154612 21635931

[36] Gassen NC Hartmann J Zschocke J Stepan J Hafner K Zellner A Association of FKBP51 with priming of autophagy pathways and mediation of antidepressant treatment response: evidence in cells, mice, and humans. PLoS Med (2014) 11:e1001755. 10.1371/journal.pmed.1001755 25386878 PMC4227651

[37] Zhang Y Liu C Zhao Y Zhang X Li B Cui R The effects of calorie restriction in depression and potential mechanisms. Curr Neuropharmacol (2015) 13:536–42. 10.2174/1570159X13666150326003852 PMC4790398 26412073

[38] He C Bassik MC Moresi V Sun K Wei Y Zou Z Exercise-induced BCL2-regulated autophagy is required for muscle glucose homeostasis. Nature (2012) 481:511–5. 10.1038/nature10758 PMC3518436 22258505

[39] Brosse AL Sheets ES Lett HS Blumenthal JA Exercise and the treatment of clinical depression in adults: recent findings and future directions 1. Sports Med (2002) 32:741–60. 10.2165/00007256-200232120-00001 12238939

[40] MacQueen GM Ramakrishnan K Ratnasingan R Chen B Young LT Desipramine treatment reduces the long-term behavioural and neurochemical sequelae of early-life maternal separation. Int J Neuropsychopharmacol (2003) 6:391–6. 10.1017/S1461145703003729 14641986

[41] Liu C Hao S Zhu M Wang Y Zhang T Yang Z Maternal separation induces different autophagic responses in the hippocampus and prefrontal cortex of adult rats. Neuroscience (2018) 374:287–94. 10.1016/j.neuroscience.2018.01.043 29391188

[42] Xia CY Wang ZZ Zhang Z Chen J Wang YY Lou YX Corticosterone impairs gap junctions in the prefrontal cortical and hippocampal astrocytes *via* different mechanisms. Neuropharmacology (2018) 131:20–30. 10.1016/j.neuropharm.2017.12.003 29223529

[43] Yang Y Hu Z Du X Davies H Huo X Fang M miR-16 and fluoxetine both reverse autophagic and apoptotic change in chronic unpredictable mild stress model rats. Front Neurosci (2017b) 11:428. 10.3389/fnins.2017.00428 28790887 PMC5524920

[44] Huang X Wu H Jiang R Sun G Shen J Ma M The antidepressant effects of a-tocopherol are related to activation of autophagy *via the* AMPK/mTOR pathway. Eur J Pharmacol (2018a) 833:1–7. 10.1016/j.ejphar.2018.05.020 29782858

[45] Zhao Z Zhang L Guo XD Cao LL Xue TF Zhao XJ Rosiglitazone exerts an anti-depressive effect in unpredictable chronic mild-stress-induced depressive mice by maintaining essential neuron autophagy and inhibiting excessive astrocytic apoptosis. Front Mol Neurosci (2017) 10:293. 10.3389/fnmol.2017.00293 28959186 PMC5603714

[46] Jiang P Guo Y Dang R Yang M Liao D Li H Salvianolic acid B protects against lipopolysaccharide-induced behavioral deficits and neuroinflammatory response: involvement of autophagy and NLRP3 inflammasome. J Neuroinflammation (2017) 14:239. 10.1186/s12974-017-1013-4 29212498 PMC5719935

[47] Levine B Liu R Dong X Zhong Q Beclin orthologs: integrative hubs of cell signaling, membrane trafficking, and physiology. Trends Cell Biol (2015a) 25:533–44. 10.1016/j.tcb.2015.05.004 PMC4554927 26071895

[48] Gulbins A Schumacher F Becker KA Wilker B Soddemann M Boldrin F Antidepressants act by inducing autophagy controlled by sphingomyelin-ceramide. Mol Psychiatry (2018). 23:2324–46. 10.1038/s41380-018-0090-9 PMC6294742 30038230

[49] Daley E Wilkie D Loesch A Hargreaves IP Kendall DA Pilkington GJ Chlorimipramine: a novel anticancer agent with a mitochondrial target. Biochem Biophys Res Commun (2005) 328:623–32. 10.1016/j.bbrc.2005.01.028 15694394

[50] Klionsky DJ Abdelmohsen K Abe A Abedin MJ Abeliovich H Acevedo AA Guidelines for the use and interpretation of assays for monitoring autophagy (3rd edition). Autophagy (2016) 12:1–222. 10.1080/15548627.2015.1100356 26799652 PMC4835977

[51] Rossi M Munarriz ER Bartesaghi S Milanese M Dinsdale D Guerra-Martin MA Desmethylclomipramine induces the accumulation of autophagy markers by blocking autophagic flux. J Cell Sci (2009) 122:3330–9. 10.1242/jcs.048181 PMC2736865 19706685

[52] Zschocke J Zimmermann N Berning B Ganal V Holsboer F Rein T Antidepressant drugs diversely affect autophagy pathways in astrocytes and neurons—dissociation from cholesterol homeostasis. Neuropsychopharmacology (2011) 36:1754–68. 10.1038/npp.2011.57 PMC3138654 21508931

[53] Zschocke J Rein T Antidepressants encounter autophagy in neural cells. Autophagy (2011) 7:1247–8. 10.4161/auto.7.10.16520 21642768

[54] Kara NZ Flaisher-Grinberg S Anderson GW Agam G Einat H Mood-stabilizing effects of rapamycin and its analog temsirolimus: relevance to autophagy. Behav Pharmacol (2018) 29:379–84. 10.1097/FBP.0000000000000334 28777104

[55] Cleary C Linde JA Hiscock KM Hadas I Belmaker RH Agam G Antidepressive-like effects of rapamycin in animal models: implications for mTOR inhibition as a new target for treatment of affective disorders. Brain Res Bull (2008) 76:469–73. 10.1016/j.brainresbull.2008.03.005 18534253

[56] Ryskalin L Limanaqi F Frati A Busceti CL Fornai F mTOR-related brain dysfunctions in neuropsychiatric disorders. Int J Mol Sci (2018) 19. 10.3390/ijms19082226 PMC6121884 30061532

[57] Motoi Y Shimada K Ishiguro K Hattori N Lithium and autophagy. ACS Chem Neurosci (2014) 5:434–42. 10.1021/cn500056q PMC4063500 24738557

[58] Pagnin D de Q Pini S Cassano GB Efficacy of ECT in depression: a meta-analytic review. J ECT (2004) 20:13–20. 10.1097/00124509-200403000-00004 15087991

[59] Otabe H Nibuya M Shimazaki K Toda H Suzuki G Nomura S Electroconvulsive seizures enhance autophagy signaling in rat hippocampus. Prog Neuropsychopharmacol Biol Psychiatry (2014) 50:37–43. 10.1016/j.pnpbp.2013.11.012 24316174

[60] Ma J Hou LN Rong ZX Liang P Fang C Li HF Antidepressant desipramine leads to C6 glioma cell autophagy: implication for the adjuvant therapy of cancer. Anticancer Agents Med Chem (2013) 13:254–60. 10.2174/1871520611313020011 22934693

[61] Sawai H Desipramine-induced lysosomal vacuolization is independent of autophagy. Cell Biol Int (2018) 42:248–53. 10.1002/cbin.10901 29068103

[62] Sundaramurthy V Barsacchi R Samusik N Marsico G Gilleron J Kalaidzidis I Integration of chemical and RNAi multiparametric profiles identifies triggers of intracellular mycobacterial killing. Cell Host Microbe (2013) 13:129–42. 10.1016/j.chom.2013.01.008 23414754

[63] Shchors K Massaras A Hanahan D Dual targeting of the autophagic regulatory circuitry in gliomas with repurposed drugs elicits cell-lethal autophagy and therapeutic benefit. Cancer Cell (2015) 28:456–71. 10.1016/j.ccell.2015.08.012 26412325

[64] Alcocer-Gómez E Casas-Barquero N Williams MR Romero-Guillena SL Canadas-Lozano D Bullon P Antidepressants induce autophagy dependent-NLRP3-inflammasome inhibition in Major depressive disorder. Pharmacol Res (2017b) 121:114–21. 10.1016/j.phrs.2017.04.028 28465217

[65] Jeon SH Kim SH Kim Y Kim YS Lim Y Lee YH The tricyclic antidepressant imipramine induces autophagic cell death in U-87MG glioma cells. Biochem Biophys Res Commun (2011) 413:311–7. 10.1016/j.bbrc.2011.08.093 21889492

[66] Cloonan SM Williams DC The antidepressants maprotiline and fluoxetine induce Type II autophagic cell death in drug-resistant Burkitt’s lymphoma. Int J Cancer (2011) 128:1712–23. 10.1002/ijc.25477 20503272

[67] Sun D Zhu L Zhao Y Jiang Y Chen L Yu Y Fluoxetine induces autophagic cell death *via* eEF2K-AMPK-mTOR-ULK complex axis in triple negative breast cancer. Cell Prolif (2018) 51:e12402. 10.1111/cpr.12402 29094413 PMC6528897

[68] Sun BK Kim JH Choi JS Hwang SJ Sung JH Fluoxetine decreases the proliferation and adipogenic differentiation of human adipose-derived stem cells. Int J Mol Sci (2015) 16:16655–68. 10.3390/ijms160716655 PMC4519971 26204837

[69] Li JR Xu HZ Nie S Peng YC Fan LF Wang ZJ Fluoxetine-enhanced autophagy ameliorates early brain injury *via* inhibition of NLRP3 inflammasome activation following subrachnoid hemorrhage in rats. J Neuroinflammation (2017) 14:186. 10.1186/s12974-017-0959-6 28903766 PMC5598033

[70] Jiang X Lu W Shen X Wang Q Lv J Liu M Repurposing sertraline sensitizes non-small cell lung cancer cells to erlotinib by inducing autophagy. JCI Insight (2018) 3. 10.1172/jci.insight.98921 PMC6124398 29875309

[71] Xia D Zhang YT Xu GP Yan WW Pan XR Tong JH Sertraline exerts its antitumor functions through both apoptosis and autophagy pathways in acute myeloid leukemia cells. Leuk Lymphoma (2017 58:1–10. 10.1080/10428194.2017.1287358 28278721

[72] Fornai F Longone P Cafaro L Kastsiuchenka O Ferrucci M Manca ML Lithium delays progression of amyotrophic lateral sclerosis. Proc Natl Acad Sci U S A (2008) 105:2052–7. 10.1073/pnas.0708022105 PMC2538879 18250315

[73] Heiseke A Aguib Y Riemer C Baier M Schatzl HM Lithium induces clearance of protease resistant prion protein in prion-infected cells by induction of autophagy. J Neurochem (2009) 109:25–34. 10.1111/j.1471-4159.2009.05906.x 19183256

[74] Fu J Shao CJ Chen FR Ng HK Chen ZP Autophagy induced by valproic acid is associated with oxidative stress in glioma cell lines. Neuro Oncol (2010) 12:328–40. 10.1093/neuonc/nop005 PMC2940599 20308311

[75] Shan Z Wei L Yu S Jiang S Ma Y Zhang C Ketamine induces reactive oxygen species and enhances autophagy in SV-HUC-1 human uroepithelial cells. J Cell Physiol (2018) 234:2778–87. 10.1002/jcp.27094 30145832

[76] Kara NZ Toker L Agam G Anderson GW Belmaker RH Einat H Trehalose induced antidepressant-like effects and autophagy enhancement in mice. Psychopharmacology (Berl) (2013) 229:367–75. 10.1007/s00213-013-3119-4 23644913

[77] Sarkar S Davies JE Huang Z Tunnacliffe A Rubinsztein DC Trehalose, a novel mTOR-independent autophagy enhancer, accelerates the clearance of mutant huntingtin and alpha-synuclein. J Biol Chem (2007) 282:5641–52. 10.1074/jbc.M609532200 17182613

[78] Li X Zhang X Zheng L Kou J Zhong Z Jiang Y Hypericin-mediated sonodynamic therapy induces autophagy and decreases lipids in THP-1 macrophage by promoting ROS-dependent nuclear translocation of TFEB. Cell Death Dis (2016b) 7:e2527. 10.1038/cddis.2016.433 28005078 PMC5260986

[79] Singh S Kumari E Bhardwaj R Kumar R Dubey VK Molecular events leading to death of *Leishmania donovani* under spermidine starvation after hypericin treatment. Chem Biol Drug Des (2017) 90:962–71. 10.1111/cbdd.13022 28509385

[80] Song X Liu B Cui L Zhou B Liu W Xu F Silibinin ameliorates anxiety/depression-like behaviors in amyloid beta-treated rats by upregulating BDNF/TrkB pathway and attenuating autophagy in hippocampus. Physiol Behav (2017) 179:487–93. 10.1016/j.physbeh.2017.07.023 28735062

[81] Yang N Li L Li Z Ni C Cao Y Liu T Protective effect of dapsone on cognitive impairment induced by propofol involves hippocampal autophagy. Neurosci Lett (2017a) 649:85–92. 10.1016/j.neulet.2017.04.019 28411068

[82] Zare N Khalifeh S Khodagholi F Shahamati SZ Motamedi F Maghsoudi N Geldanamycin reduces Abeta-associated anxiety and depression, concurrent with autophagy provocation. J Mol Neurosci (2015) 57:317–24. 10.1007/s12031-015-0619-1 26208597

[83] Huang Z Huang X Wang Q Jiang R Sun G Xu Y Extract of Euryale ferox Salisb exerts antidepressant effects and regulates autophagy through the adenosine monophosphate-activated protein kinase-UNC-51-like kinase 1 pathway. IUBMB Life (2018b) 70:300–9. 10.1002/iub.1731 29509332

[84] Fries GR Gassen NC Rein T The FKBP51 Glucocorticoid receptor co-chaperone: regulation, function, and implications in health and disease. Int J Mol Sci (2017) 18. 10.3390/ijms18122614 PMC5751217 29206196

[85] Wochnik GM Rüegg J Abel GA Schmidt U Holsboer F Rein T FK506-binding proteins 51 and 52 differentially regulate dynein interaction and nuclear translocation of the glucocorticoid receptor in mammalian cells. J Biol Chem (2005) 280:4609–16. 10.1074/jbc.M407498200 15591061

[86] Rein T FK506 binding protein 51 integrates pathways of adaptation: FKBP51 shapes the reactivity to environmental change. Bioessays (2016) 38:894–902. 10.1002/bies.201600050 27374865

[87] Gassen NC Hartmann J Schmidt MV Rein T FKBP5/FKBP51 enhances autophagy to synergize with antidepressant action. Autophagy (2015) 11:578–80. 10.1080/15548627.2015.1017224 PMC4502647 25714272

[88] Gulbins E Palmada M Reichel M Luth A Bohmer C Amato D Acid sphingomyelinase-ceramide system mediates effects of antidepressant drugs. Nat Med (2013) 19:934–8. 10.1038/nm.3214 23770692

[89] Kornhuber J Tripal P Reichel M Terfloth L Bleich S Wiltfang J Identification of new functional inhibitors of acid sphingomyelinase using a structure-property-activity relation model. J Med Chem (2008) 51:219–37. 10.1021/jm070524a 18027916

[90] Pasquali L Busceti CL Fulceri F Paparelli A Fornai F Intracellular pathways underlying the effects of lithium. Behav Pharmacol (2010) 21:473–92. 10.1097/FBP.0b013e32833da5da 20700048

[91] Sarkar S Floto RA Berger Z Imarisio S Cordenier A Pasco M Lithium induces autophagy by inhibiting inositol monophosphatase. J Cell Biol (2005) 170:1101–11. 10.1083/jcb.200504035 PMC2171537 16186256

[92] Kirmeier T Gopalakrishnan R Gormanns V Werner AM Cuboni S Rudolf GC Azidobupramine, an antidepressant-derived bifunctional neurotransmitter transporter ligand allowing covalent labeling and attachment of fluorophores. PLoS One (2016) 11:e0148608. 10.1371/journal.pone.0148608 26863431 PMC4749225

[93] Eliwa H Belzung C Surget A Adult hippocampal neurogenesis: is it the alpha and omega of antidepressant action? Biochem Pharmacol (2017) 141:86–99. 10.1016/j.bcp.2017.08.005 28800956

[94] Wu X Fleming A Ricketts T Pavel M Virgin H Menzies FM Autophagy regulates Notch degradation and modulates stem cell development and neurogenesis 1. Nat Commun (2016) 7:10533. 10.1038/ncomms10533 26837467 PMC4742842

[95] Yazdankhah M Farioli-Vecchioli S Tonchev AB Stoykova A Cecconi F The autophagy regulators Ambra1 and Beclin 1 are required for adult neurogenesis in the brain subventricular zone. Cell Death Dis (2014) 5:e1403. 10.1038/cddis.2014.358 25188513 PMC4540193

[96] Fornai F Ferrucci M Lenzi P Falleni A Biagioni F Flaibani M Plastic changes in the spinal cord in motor neuron disease. Biomed. Res Int (2014) 2014:670756. 10.1155/2014/670756 24829911 PMC4009217

[97] Rein T Is autophagy involved in the diverse effects of antidepressants? Cells (2019) 8. 10.3390/cells8010044 PMC6356221 30642024

[98] Zanos P Gould TD Mechanisms of ketamine action as an antidepressant. Mol Psychiatry (2018) 23:801–11. 10.1038/mp.2017.255 PMC5999402 29532791

[99] Glotzbach RK Preskorn SH Brain concentrations of tricyclic antidepressants: single-dose kinetics and relationship to plasma concentrations in chronically dosed rats. Psychopharmacology (Berl) (1982) 78:25–7. 10.1007/BF00470582 6815692

[100] Holladay JW Dewey MJ Yoo SD Pharmacokinetics and antidepressant activity of fluoxetine in transgenic mice with elevated serum alpha-1-acid glycoprotein levels. Drug Metab Dispos (1998) 26:20–4. 9443847

[101] Hiemke C Baumann P Bergemann N Conca A Dietmaier O Egberts K AGNP consensus guidelines for therapeutic drug monitoring in psychiatry: update 2011. Pharmacopsychiatry (2011) 44:195–235. 10.1055/s-0031-1286287 21969060

[102] Shehata M Matsumura H Okubo-Suzuki R Ohkawa N Inokuchi K Neuronal stimulation induces autophagy in hippocampal neurons that is involved in AMPA receptor degradation after chemical long-term depression. J Neurosci (2012) 32:10413–22. 10.1523/JNEUROSCI.4533-11.2012 PMC6703735 22836274

[103] Hernandez D Torres CA Setlik W Cebrian C Mosharov EV Tang G Regulation of presynaptic neurotransmission by macroautophagy. Neuron (2012) 74:277–84. 10.1016/j.neuron.2012.02.020 PMC3578406 22542182

[104] Uytterhoeven V Lauwers E Maes I Miskiewicz K Melo MN Swerts J Hsc70-4 deforms membranes to promote synaptic protein turnover by endosomal microautophagy. Neuron (2015) 88:735–48. 10.1016/j.neuron.2015.10.012 26590345

[105] McPherson PS Eating locally: microautophagy and protein turnover at the synapse. Neuron (2015) 88:619–21. 10.1016/j.neuron.2015.11.008 26590336

[106] Li D Zheng J Wang M Feng L Ren Z Liu Y Changes of TSPO-mediated mitophagy signaling pathway in learned helplessness mice. Psychiatry Res (2016a) 245:141–7. 10.1016/j.psychres.2016.02.068 27543827

[107] Limanaqi F Biagioni F Gambardella S Ryskalin L Fornai F Interdependency between autophagy and synaptic vesicle trafficking: implications for dopamine release. Front Mol Neurosci (2018) 11:299. 10.3389/fnmol.2018.00299 30186112 PMC6110820

[108] Mu Y Yan X Li D Zhao D Wang L Wang X NUPR1 maintains autolysosomal efflux by activating SNAP25 transcription in cancer cells. Autophagy (2018) 14:654–70. 10.1080/15548627.2017.1338556 PMC5959327 29130426

[109] Enomoto S Shimizu K Nibuya M Toda H Yoshino A Suzuki E Increased expression of endocytosis-related proteins in rat hippocampus following 10-day electroconvulsive seizure treatment. Neurosci Lett (2016) 624:85–91. 10.1016/j.neulet.2016.05.015 27177725

